# FcγR-TLR Cross-Talk Enhances TNF Production by Human Monocyte-Derived DCs via IRF5-Dependent Gene Transcription and Glycolytic Reprogramming

**DOI:** 10.3389/fimmu.2019.00739

**Published:** 2019-04-08

**Authors:** Willianne Hoepel, Melissa Newling, Lisa T. C. Vogelpoel, Lathees Sritharan, Ivo S. Hansen, Martien L. Kapsenberg, Dominique L. P. Baeten, Bart Everts, Jeroen den Dunnen

**Affiliations:** ^1^Amsterdam Rheumatology and Immunology Center, Amsterdam, Netherlands; ^2^Department of Experimental Immunology, Amsterdam UMC, Amsterdam Infection and Immunity Institute, University of Amsterdam, Amsterdam, Netherlands; ^3^Department of Parasitology, Leiden University Medical Center, Leiden, Netherlands

**Keywords:** Fc gamma receptor (FcγR), interferon regulatory factor 5 (IRF5), dendritic cells, macrophages, glycolytic reprogramming, tumor necrosis factor (TNF), rheumatoid arthritis (RA), chronic inflammation

## Abstract

Antigen-presenting cells (APCs) such as dendritic cells (DCs) are crucial for initiation of adequate inflammatory responses, which critically depends on the cooperated engagement of different receptors. In addition to pattern recognition receptors (PRRs), Fc gamma receptors (FcγRs) have recently been identified to be important in induction of inflammation by DCs. FcγRs that recognize IgG immune complexes, which are formed upon opsonization of pathogens, induce pro-inflammatory cytokine production through cross-talk with PRRs such as Toll-like receptors (TLRs). While the physiological function of FcγR-TLR cross-talk is to provide protective immunity against invading pathogens, undesired activation of FcγR-TLR cross-talk, e.g., by autoantibodies, also plays a major role in the development of chronic inflammatory disorders such as rheumatoid arthritis (RA). Yet, the molecular mechanisms of FcγR-TLR cross-talk are still largely unknown. Here, we identified that FcγR-TLR cross-talk-induced cytokine production critically depends on activation of the transcription factor interferon regulatory factor 5 (IRF5), which results from induction of two different pathways that converge on IRF5 activation. First, TLR stimulation induced phosphorylation of TBK1/IKKε, which is required for IRF5 phosphorylation and subsequent activation. Second, FcγR stimulation induced nuclear translocation of IRF5, which is essential for gene transcription by IRF5. We identified that IRF5 activation by FcγR-TLR cross-talk amplifies pro-inflammatory cytokine production by increasing cytokine gene transcription, but also by synergistically inducing glycolytic reprogramming, which is another essential process for induction of inflammatory responses by DCs. Combined, here we identified IRF5 as a pivotal component of FcγR-TLR cross-talk in human APCs. These data may provide new potential targets to suppress chronic inflammation in autoantibody-associated diseases that are characterized by undesired or excessive FcγR-TLR cross-talk, such as RA, systemic sclerosis, and systemic lupus erythematous.

## Introduction

Protection against different classes of pathogens requires the activation of antigen-presenting cells (APCs) such as dendritic cells (DCs). A crucial step for shaping both innate and adaptive immunity by DCs is the production of various pro-inflammatory cytokines. DCs produce these cytokines upon detection of pathogens or endogenous danger signals via activation of different families of receptors, which collectively are referred to as pattern recognition receptors (PRRs). Well-known examples of PRRs include the families of Toll-like receptors (TLRs), NOD-like receptors (NLRs), C-type lectins, and RIG-I-like receptors (RLRs). However, the list of receptor families that control cytokine production is still expanding.

In recent years, it has become clear that also the family of Fc gamma receptors (FcγRs), which are receptors for the Fc region of immunoglobulin G (IgG), play an important role in the induction of cytokines by DCs. While individual stimulation of FcγRs elicits little cytokine production, FcγRs synergize with PRRs such as TLRs to strongly but selectively amplify pro-inflammatory cytokine production. FcγRs synergize with TLRs that are expressed both intracellular (TLR3, TLR7/8) and extracellular (TLR2, TLR4, TLR5), as well as other receptors such as NLRs and particular cytokine receptors (1, 2). Combined, modulation of cytokine production by FcγRs thereby tailors immune responses to the immunological context (3, 4).

In human APCs such as DCs, the best studied cytokine-inducing FcγR is FcγRIIa. FcγRIIa has a low affinity for IgG, and is therefore able to discriminate between unbound IgG and IgG immune complexes (i.e., antigen-bound). While unbound IgG, as present under homeostatic conditions, induces inhibitory signaling (5), stimulation of FcγRIIa with immune complexes, as present on opsonized pathogens, strongly enhances cytokine production induced by TLRs (1, 6). Although monocytes and macrophages are known also to express other FcRs such as FcγRI, FcγRIIa is the main IgG receptor responsible for amplifying TLR responses (2).

The physiological function of FcγR-TLR cross-talk is to counteract infections with various classes of pathogens. For example, upon IgG opsonization of bacteria, the simultaneous activation of FcγRIIa and TLRs specifically amplifies the production of pro-inflammatory cytokines TNF, IL-1β, IL-6, and IL-23 by human DCs, which in turn promote human T helper 17 (Th17) skewing, thereby tailoring immune response to counteract extracellular bacterial infections (1, 6). However, in addition to its physiological function, FcγR-TLR cross-talk can also be induced undesirably by immune complex formation of autoantibodies. This pathological role of FcγR-TLR cross-talk contributes to the pathogenesis of various autoimmune diseases including rheumatoid arthritis (RA) (7).

Remarkably, while FcγR-induced cytokine production plays an important role in both host defense and various autoimmune diseases, still very little is known about the underlying molecular mechanisms. Similar to other FcγR-mediated functions such as phagocytosis and ADCC, FcγR-induced cytokine production is dependent on the upstream kinase Syk (8). However, recent findings indicate that the downstream signaling events required for FcγR-induced cytokine production are distinct from other FcγR-mediated functions such as phagocytosis (3). Compared to FcγR signaling, relatively more is known about the signaling pathways that are induced by individual stimulation of TLRs. TLRs signal via adaptor proteins such as MyD88 and/or TRIF to activate various transcription factors including NF-κB and MAP kinases, which are important for the transcription of pro-inflammatory cytokines such as TNF (9). Yet, how TLR and FcγR signaling pathways collaborate to synergistically amplify pro-inflammatory cytokine production is still largely unknown.

In this study, we identified that FcγR-TLR cross-talk-induced cytokine production critically depends on activation of the transcription factor interferon regulatory factor 5 (IRF5), which results from collaborative IRF5 activation by both FcγRs and TLRs. While TLR stimulation induced IRF5 phosphorylation, FcγR stimulation was required for IRF5 nuclear translocation. Moreover, we identified that IRF5 activation by FcγR-TLR cross-talk amplified pro-inflammatory cytokines production by both increasing cytokine gene transcription and by inducing glycolytic reprogramming, thereby identifying FcγRs as a new family of receptors that can induce metabolic reprogramming.

## Materials and Methods

### Cells and Stimulation

This study was done according to the ethical guidelines of the Academic Medical Center and human material was obtained in accordance with the AMC Medical Ethics Review Committee according to the Medical Research Involving Human Subjects Act. Buffy coats obtained after blood donation (Sanquin blood supply) are not subjected to informed consent, which is according to the Medical Research Involving Human Subjects Act and the AMC Medical Ethics Review Committee. All samples were handled anonymously. Ethical review and approval was not required for this study in accordance with the local legislation. Monocytes were isolated from buffy coats by density gradient centrifugation on Lymphoprep (Nycomed) and Percoll (Pharmacia). DCs or macrophages were generated by culturing monocytes for 6 days in IMDM (Lonza) containing 5% FBS (Biowest) and 86 μg/mL gentamicin (Gibco), supplemented with 20 ng/mL GM-CSF (Invitrogen) and 2 ng/mL IL-4 (Miltenyi Biotec) for DCs or 50 ng/mL recombinant human M-CSF (BioLegend) for macrophages. At day 2 or 3, half of the medium was replaced by new medium containing cytokines.

For silencing at day 3, cells were harvest by resuspending (DCs) or by using TrypLE Select (Invitrogen) (macrophages). Cells were microporated in the presence of 500 nM IRF5 si-RNA or control si-RNA (Dharmacon) and cultured for 3 more days in IMDM without gentamicin with supplemented cytokines.

DCs were harvested at day 6 by putting the cells on ice for 30 min and macrophages were harvested at day 6 by TrypLE Select. For cIgG stimulation, 96-well high-affinity Maxisorp plates (Nunc) were coated with 2 μg/mL IgG from pooled IgG (Nanogam; Sanquin Blood Supply) diluted in PBS overnight at 4°C, followed by blocking with PBS containing 10% FBS for 1 h at 37°C. Cells were stimulated (30,000–50,000 cells per well) with 10 μg/mL Pam3CSK4 (Invivogen). Co-stimulation experiments were performed by simultaneous exposure of the cells to cIgG and Pam3. Syk was inhibited with 1 μM R406 (Selleckchem), TBK1/IKKε was inhibited with 2 μM BX795 (Invivogen) and glycolysis was blocked using 10 mM 2-Deoxy-D-glucose (2DG; Sigma Aldrich). Cells were incubated with the inhibitor or the corresponding volume of DMSO (Sigma-Aldrich) or medium for 30 min at 37°C before stimulation.

### Quantitative RT-PCR

For mRNA-level analysis, cells were lysed at the indicated time points, after which mRNA extraction was performed using RNeasy Mini Kit (Qiagen) and cDNA synthesis using RevertAid H Minus First Strand cDNA Synthesis Kit (Fermentas). Quantitative RT-PCR (StepOnePlus Real-Time PCR System; Thermo Fisher Scientific) was performed using Taqman Master Mix and the following Taqman primers (all from Thermo Fisher Scientific): *GAPDH* (4310884E), *IRF5* (Hs00158114_m1), and *TNF* (Hs00174128_m1). mRNA levels were normalized to the geometric mean of the Ct-values of housekeeping gene *GAPDH* [2^Ct(housekeeping)−Ct(target)^], and folds were calculated compared with an unstimulated control sample (*t* = 0 h).

### ELISA

For analysis of cytokine production, supernatants were harvested after overnight stimulation and stored at −20°C. Cytokine levels in supernatants were measured by ELISA, using antibody pairs for TNF (eBioscience), IL-Iβ, IL-6, and IL-23 (U-CyTech Biosciences).

### Fluorescence Microscopy

For analysis of IRF5 translocation, DCs or macrophages were stimulated as indicated in Maxisorp plates. After 2 h stimulation, cells were washed with PBS, fixed with 3.7% formaldehyde (Sigma-Aldrich) for 15 min at room temperature, washed in PBS and stored in PBS containing 0.5% bovine serum albumin (BSA; PAA) and 0.1% sodium azide (Merck) at 4°C. Cells were permeabilized with 0.2% Triton X-100 (Sigma-Aldrich) for 5 min at room temperature and blocked for 30 min in PBS containing 0.5% BSA and 0.1% sodium azide. Cells were then stained with a rabbit-anti-human-IRF5 antibody (1:400) (Cell Signaling) or rabbit-anti-human NF-kB p65 antibody (1:100) (Cell Signaling) for 45 min at room temperature, washed with PBS and stained with a Cy3-labeled goat-anti-rabbit-IgG antibody (1:50) (Jackson ImmunoResearch). Cells were again washed with PBS and nuclei were stained using 1 μg/mL Hoechst (Immunochemistry Technologies) for 1 min at room temperature. Cells were imaged using a DM IRB inverted fluorescence microscope (Leica), combined with a DFC 300FX digital color camera (Leica).

### Flow Cytometry

For analysis of TBK1/IKKε phosphorylation, DCs or macrophages were stimulated as indicated in 48-well plates (Greiner Bio-One) for 30 min and fixed using Lyse/Fix buffer (BD Biosciences) for 10 min at room temperature. For analysis of IRF5, unstimulated DCs were also lysed and transferred in a 96-well plate following the same protocol as TBK1 phosphorylation. Cells were harvested by gentle scraping, transferred to a 96-well round-bottom plate (Greiner Bio-One), washed in PBS, and permeabilized using Perm III buffer (BD Biosciences) for at least 30 min at −20°C. Cells were then washed in PBS containing 0.5% BSA and 0.1% sodium azide and stained for 1 h at RT with a rabbit-anti-human-IRF5 antibody (1:200) (Cell Signaling) or a rabbit-anti-human-pTBK1 antibody (1:50) (Ser172; Cell Signaling), which also reacts to pIKKε, followed by a 30 min staining at room temperature with Alexafluor488-labeled goat-anti-rabbit-IgG antibody (1:400) (Molecular Probes). Fluorescence was determined by flow cytometry (Canto II, BD Biosciences).

### Metabolic Assays

Real-time analysis of the extracellular acidification rate (ECAR) and the oxygen consumption rate (OCR) of DCs were analyzed using an XF-96 Extracellular Flux Analyzer (Seahorse Bioscience). 30,000 DCs were plated per well. To trigger FcγR on DCs XF-96 cell culture plates were coated with 4 μg/ml IgG prior to seeding of the cells. DCs were plated in glucose-free medium after which glucose was added (10 mM) to the cells during the assay to be able to determine true glycolysis-driven ECAR. Thirty minutes after glucose addition cells were stimulated with 10 μg/mL Pam3CSK4 during the essay after which OCR and glycolysis-driven ECAR were determined 30 min post stimulation.

### Western Blot

For analysis of IRF5 phosphorylation, DCs were stimulated as indicated in 6-well plates (1,250,000–2,000,000 cell per well) (Costar) for 30 min. Cells were gently scraped and collected in cold PBS. After washing, cells were lysed on ice for 10 min using RIPA lysis buffy (Cell signaling) supplemented with protease and phosphatase inhibitors (both from Roche). Lysates were briefly sonificiated for 10 s at 30% and centrifuged for 10 min at 14,000 × g. BCA assay was performed (Thermo Scientific) and samples were boiled with 4x Laemmli Sample Buffer (Bio-Rad) for 15 min at 95°C. Cell lysates were run on a 4–12% Bis-Tris protein gel (Invitrogen) using MES-running buffer (Invitrogen). Proteins were transferred to a PVDF membrane (GE healthcare) using transfer buffer (Invitrogen) and blocked with 2% milk (Bio-Rad) afterwards. Membrane was incubated in TBS Tween o/n at 4°C with indicated antibodies: Phospho-IRF5 (Ser437) polyclonal antibody (1:1000) (Thermo Scientific), IRF5 (1:1000) (E1N9G) rabbit mAb (Cell Signaling), or Actin antibody (I-19) (1:2000) (Santa Cruz). Afterwards membrane washed with TBS Tween and incubated for 1 h at room temperature with polyclonal swine anti-rabbit immunoglobulins HRP (1:3000) (Dako).

### Data Analysis

Co-localization quantification of the fluorescence microscopy data was done using Huygens Professional software (SVI, Hilversum, The Netherlands) calculating the Manders Coefficients. Western blots were analyzed using ImageJ. Data were analyzed for statistical significance using student's *t*-test with GraphPad Prism version 7 software (GraphPad Software).

## Results

### FcγR-TLR Cross-Talk in Human moDCs and Macrophages Is Dependent on IRF5

**Figure 1:**
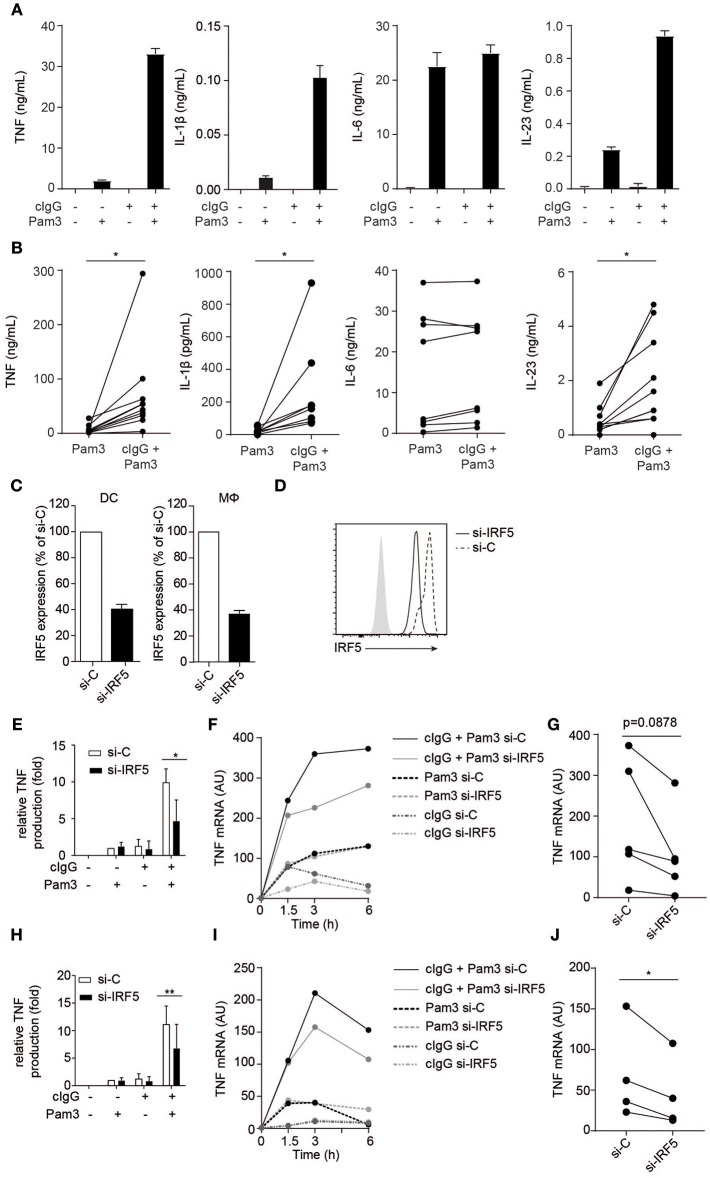
FcγR-TLR cross-talk in human moDCs and macrophages is dependent on IRF5. **(A,B)** Human monocyte-derived dendritic cells (moDC) were stimulated with Pam3CSK4 (Pam3), cIgG, or the combination for 24 h. Protein production was determined by ELISA. **(A)** Representative examples of experiments performed in triplicate (mean + SEM). **(B)** Protein production of multiple donors, each pair of dots represent one donor. **(C,D)** IRF5 in human moDCs and macrophages was silenced using specific si-RNA. **(C)** *IRF5* mRNA expression of unstimulated moDCs or macrophages (Mϕ), after IRF5 silencing (si-IRF5) or non-targeted control silencing (si-C). Data shown is *IRF5* mRNA expression as percentage of control of *IRF5* mRNA expression in si-C conditions. Mean + SD of three (moDC) or eight experiments (Mϕ). **(D)** IRF5 protein expression of unstimulated moDCs after IRF5 silencing or non-targeted control silencing measured by flow cytometry. **(E,H)** Control or IRF5-silenced moDCs **(E)** and macrophages **(H)** were stimulated with Pam3, cIgG, or the combination for 6 h. Protein production was determined by ELISA. Data shown is protein production normalized to Pam3-induced TNF production for each experiment (set to 1), mean + SD of three **(E)** or six **(H)** experiments using different donors. **(F,I)** Control or IRF5-silenced moDCs **(F)** and macrophages **(I)** were stimulated with Pam3, cIgG, or in combination and *TNF* mRNA expression (normalized to housekeeping gene expression) was determined at indicated time points by quantitative RT-PCR. Representative examples of four experiments. **(G,J)** *TNF* mRNA expression after 6 h co-stimulation of control or IRF5-silenced moDCs **(G)** and Mϕ **(J)** of multiple donors. Each pair of dots represents one donor. ^*^p < 0.05, ^**^p < 0.01, Student's *t*-test.

FcγR-TLR cross-talk plays an important role in inducing inflammation during both bacterial infections and autoimmune diseases (1, 2, 6, 8). As illustrated in Figures 1A,B (representative donor and multiple donors, respectively), FcγR-TLR cross-talk synergistically amplifies the production of key pro-inflammatory cytokines TNF, IL-1β, and IL-23, while other cytokines such as IL-6 are not affected. Here, we set out to identify the molecular mechanisms underlying this response, using TNF production as a main read-out for FcγR-TLR cross-talk. FcγR-TLR cross-talk is known to amplify TNF production at the level of gene transcription (1, 8). Here, we hypothesized a role for IRF5, since this transcription factor is known to be involved in enhancing *TNF* transcription (10–14), is highly expressed in human myeloid APCs (15), and since *IRF5* polymorphisms are a known risk factor for several autoimmune diseases (16–22).

To study the role of IRF5 in FcγR-TLR cross-talk, we made use of a small interfering (si)-RNA approach, which on average resulted in a 60 % reduction of *IRF5* mRNA expression and a similar reduction in IRF5 protein in monocyte-derived DCs (moDCs) (Figures 1C,D). For stimulation of FcγRs and TLR2 we used plate-bound complexed IgG (cIgG) and Pam3CSK4 (Pam3), respectively. While individual stimulation with cIgG or Pam3 induced moderate amounts of TNF, combined stimulation strongly and synergistically amplified TNF production (Figure 1E). However, strikingly, silencing of *IRF5* specifically reduced TNF protein production by FcγR-TLR cross-talk, without affecting cytokine production induced by the individual ligands (Figure 1E). In addition, we assessed whether IRF5 is also responsible for FcγR-TLR cross talk-induced gene transcription. Indeed, (partial) silencing of IRF5 reduced *TNF* mRNA production upon FcγR-TLR co-stimulation (Figure 1F for kinetics of representative donor, Figure 1G for multiple donors). In contrast, *TNF* mRNA induced by TLR stimulation alone was not affected by IRF5 silencing (Figure 1F), indicating that IRF5 specifically controls *TNF* transcription induced by FcγR-TLR cross-talk.

To determine whether IRF5 is only essential for FcγR-TLR cross-talk in moDCs, or whether it is also required for FcγR-TLR cross-talk in other cell types, we assessed the effect of IRF5 silencing on human macrophages, which are the main source of TNF in inflamed synovia of RA patients (23). Similar to moDCs, silencing of IRF5 in monocyte-derived macrophages (Figure 1C) specifically reduced TNF production induced by FcγR-TLR synergy, both on protein (Figure 1H) and mRNA (Figures 1I,J).

Combined, these data demonstrate that the synergistic induction of TNF by FcγR-TLR cross-talk in human moDCs and macrophages is dependent on IRF5.

### FcγR Stimulation Induces IRF5 Nuclear Translocation

**Figure 2:**
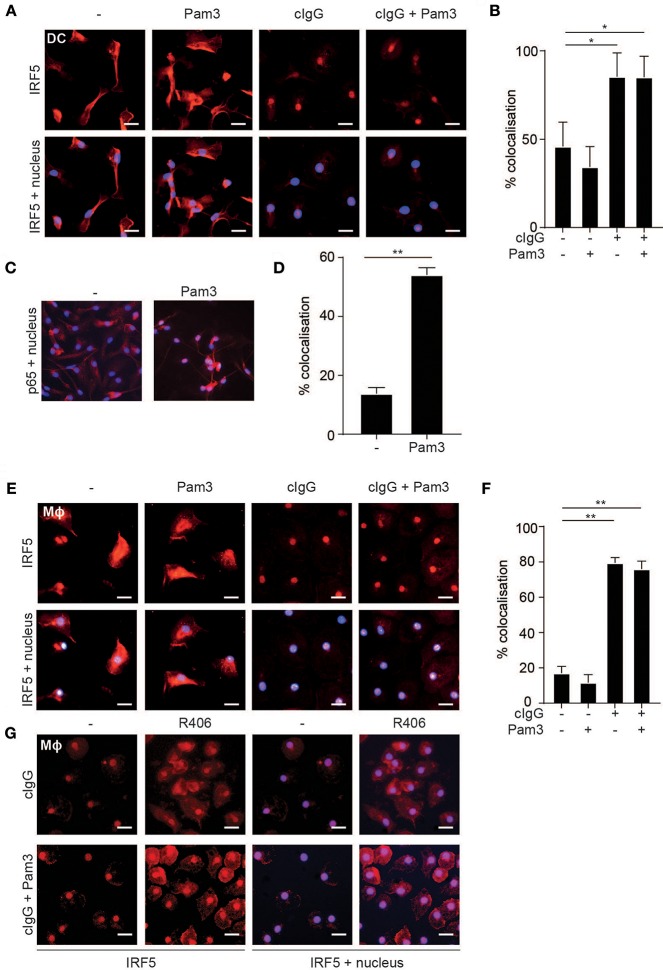
FcγR stimulation induces IRF5 nuclear translocation. **(A,E)** Human monocyte-derived dendritic cells (moDC) **(A)** and macrophages (Mϕ) **(E)** were stimulated with Pam3CSK4 (Pam3), cIgG, or the combination for 2 h and stained for IRF5 (red) and nuclei (Hoechst, blue). Representative images of three independent experiments, bar: 20 μm. **(B,D,F)** Quantification of the microscopy data showing percent co-localization of three experiments (mean + SD). ^*^p < 0.05, ^**^p < 0.01, Student's *t*-test. **(C)** moDCs were stimulated with Pam3 and stained for p65 (red) and nuclei (Hoechst, blue). Representative image of three independent experiments. **(G)** Human monocyte derived macrophages were pre-incubated with the Syk inhibitor R406 and stimulated with Pam3, cIgG, or the combination for 2 h and stained for IRF5 (red) and nuclei (Hoechst, blue). Representative images of three independent experiments, bar: 20 μm.

The transcription factor IRF5 is constitutively expressed by myeloid APCs (15), but to regulate gene transcription IRF5 needs to be translocated to the nucleus (24). Therefore, we assessed IRF5 localization in human moDCs by fluorescence microscopy upon FcγR-TLR co-stimulation. IRF5 contains two nuclear localization signals (NLS) as well as a nuclear export signal (NES) and therefore continuously shuttles in and out of the nucleus (25–27). Indeed, in unstimulated moDCs IRF5 was present throughout the cell, both in the nucleus and the cytoplasm (Figure 2A). Similar to unstimulated cells, TLR2-stimulated moDCs also displayed an even distribution of IRF5 (Figure 2A). In contrast, stimulation with cIgG, either combined with TLR stimulation or not, resulted in near exclusive accumulation of IRF5 in the nucleus (Figure 2A; quantified in Figure 2B). As a control we also ascertained that (individual) TLR stimulation of moDCs results in nuclear translocation of NF-κB subunit p65 (Figures 2C,D), which is responsible for TLR-induced pro-inflammatory cytokine production. Very similar to moDCs, FcγR stimulation induced IRF5 nuclear translocation in human macrophages (Figures 2E,F), suggesting that nuclear translocation of IRF5 induced by FcγR stimulation is a general mechanism in myeloid APCs.

Since FcγR-TLR cross-talk is known to depend on signaling through the kinase Syk, we next assessed whether Syk is required for IRF5 nuclear translocation. As shown in Figure 2G, Syk inhibition by therapeutic small-molecule inhibitor R406 indeed suppressed IRF5 nuclear translocation both upon individual stimulation with cIgG and upon cIgG+Pam3 co-stimulation.

These data indicate that, in human moDCs and macrophages, stimulation with IgG immune complexes is responsible for nuclear translocation of IRF5.

### FcγR-TLR Cross-Talk Is Dependent on TLR-Induced Phosphorylation of TBK1/IKKε and IRF5

**Figure 3:**
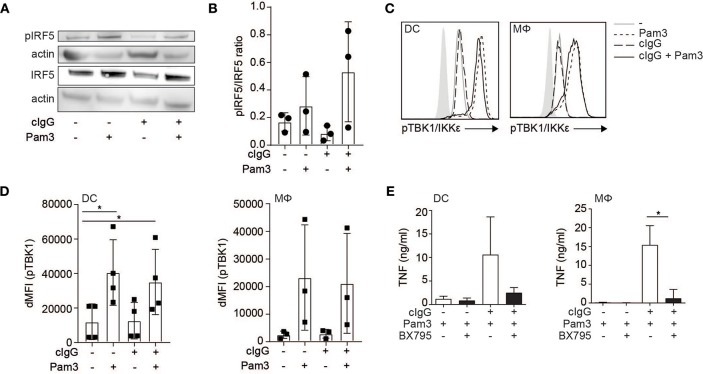
FcγR-TLR cross-talk is dependent on TLR-induced phosphorylation of TBK1/IKKε and IRF5. **(A)** Human monocyte-derived dendritic cells (moDC) were stimulated with Pam3CSK4 (Pam3), cIgG, or the combination for 30 min. IRF5 phosphorylation at Ser437 and total IRF5 expression was assessed by Western blot. Data shown is representative example of three independent experiments. **(B)** Quantification of the three independent Western blot experiments using ImageJ (mean + SD). First bands were corrected for actin, after which the pIRF5/IRF5 ratio was calculated. **(C)** Human moDCs and macrophages (Mϕ) were stimulated with Pam3, cIgG, or the combination for 30 min and stained for p-TBK1/IKKε and analyzed by flow cytometry (^10^log scale, light gray indicates background staining). Representative example of four (DCs) and three (Mϕ) experiments. **(D)** ΔMFI of pTBK1 of four (moDCs) and three (Mϕ) independent experiments (mean + SD). **(E)** After pre-incubation with 2 μM BX795 or the corresponding volume of DMSO, moDCs, and macrophages were stimulated with Pam3, or cIgG combined with Pam3 for 24 h and TNF production was determined by ELISA. Mean + SD of four independent experiments. ^*^p < 0.05, Student's *t*-test.

While individual FcγR stimulation induced IRF5 translocation into the nucleus, it is not sufficient to induce *TNF* transcription (1, 6, 8). Importantly, in addition to nuclear translocation, IRF5 needs to be activated by phosphorylation in order to be transcriptionally active (14, 25, 26, 28, 29). Therefore, we determined IRF5 phosphorylation upon (co-)stimulation of human moDCs by Western blot. Interestingly, while Pam3 stimulation induced IRF5 phosphorylation, stimulation with cIgG did not (Figure 3A, quantified as pIRF5/IRF5 ratio for multiple donors in Figure 3B). These data indicate that while FcγR stimulation induces IRF5 nuclear translocation, TLR stimulation is required for IRF5 phosphorylation.

IRF5 phosphorylation can be induced by TBK1, a member of the Iκ kinase (IKK) family that shares larges structural and functional similarity to IKKε (25, 26, 30). Since TBK1/IKKε also needs to be phosphorylated in order to execute kinase activity (31), we assessed TBK1/IKKε phosphorylation by flow cytometry after (co-)stimulation. Similar to IRF5 phosphorylation, we found that stimulation with Pam3 induced TBK1/IKKε phosphorylation, while stimulation with cIgG did not (Figure 3C, quantified for multiple donors in Figure 3D).

To determine whether TBK1/IKKε is required for cytokine production by FcγR-TLR cross-talk, we inhibited TBK1/IKKε using small-molecule inhibitor BX795. Indeed, BX795 abrogated FcγR-TLR cross-talk-induced TNF production (Figure 3E).

Thus, while FcγR stimulation induces nuclear translocation of IRF5, TLR stimulation induces phosphorylation of TBK1/IKKε and IRF5, which combined results in nuclear translocation of phosphorylated IRF5 to modulate cytokine gene transcription.

### FcγR-TLR Cross-Talk Induces Glycolytic Reprogramming via IRF5

**Figure 4:**
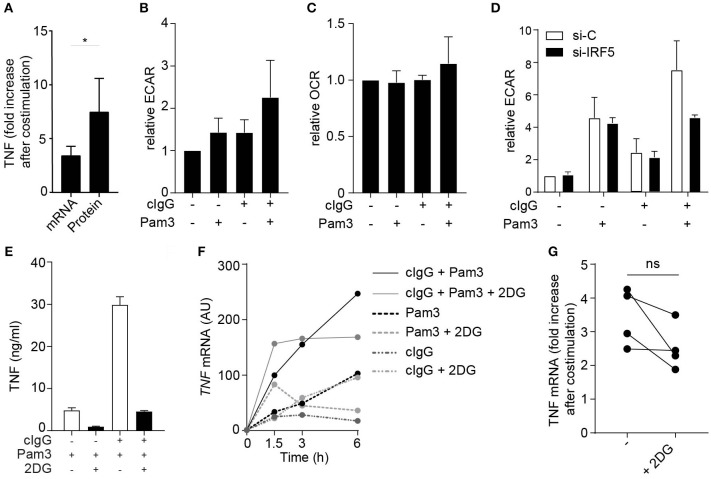
FcγR-TLR cross-talk induces glycolytic reprogramming via IRF5. **(A)** TNF fold increase in human monocyte derived DCs (moDCs) after co-stimulation with Pam3 and cIgG for mRNA and protein. Fold increase of TNF was determined by setting Pam3 stimulation at 1 and calculating fold increase after co-stimulation at *t* = 3 h (mRNA) or *t* = 24 h (protein). Mean + SD of four (mRNA) or eight (protein) experiments. **(B,C)** moDCs were stimulated for 30 min with Pam3, cIgG, or the combination and extracellular acidification rate (ECAR) **(B)** and oxygen consumption rate (OCR) **(C)** was determined. Values are normalized to unstimulated moDCs for each experiment (set to 1). Mean + SD of four experiments. **(D)** Control or IRF5-silenced moDCs were stimulated with Pam3, cIgG, or the combination for 30 min and ECAR was measured. Representative experiment in triplicate of three independent experiments. **(E,F)** After 30 min pre-incubation with 10 mM 2-Deoxy-D-glucose (2DG), moDCs were stimulated with Pam3, cIgG, or in combination. **(E)** TNF production after 24 h was determined by ELISA; representative example in triplicate of eight independent experiments. **(F)** *TNF* mRNA expression was determined at indicated time points (normalized to housekeeping gene expression) by quantitative RT-PCR; representative example of four independent experiments. **(G)** TNF fold increase after co-stimulation with Pam3 and cIgG with and without 2DG. Fold increase of TNF was determined by setting Pam3 stimulation at 1 and calculating fold increase after co-stimulation at *t* = 3 h. Mean + SD of four experiments. Each pair of dots represent one donor. ^*^p < 0.05, Student's *t*-test.

Amplification of cytokine production can be orchestrated at both the transcriptional and translational level. Interestingly, upon FcγR co-stimulation of moDCs, the fold increase in expression of *TNF* mRNA was lower than that fold increase at the protein level (Figure 4A), suggesting that increased translation also contributes to the amplified cytokine response. In DCs, increased cytokine mRNA translation in response to TLR stimulation has been shown to be underpinned by a rapid increase in glycolytic rate, to serve as a carbon source for *de novo* fatty acid synthesis to support expansion of the endoplasmic reticulum required for increased cytokine gene translation (32, 33). This, together with the recent finding that IRF5 is able to increase the glycolysis in macrophages (34), led us to hypothesize that FcγR (co-) stimulation induces a similar metabolic reprogramming via IRF5 to support increased translation. To this end, we stimulated moDCs and analyzed them for changes in rates of extracellular acidification (ECAR), as a measure of lactate production (a proxy for the glycolytic rate), and the rate of oxygen consumption (OCR), as a measure of oxidative phosphorylation. Notably, stimulation with cIgG indeed increased the ECAR, which was even further enhanced upon co-stimulation with cIgG and Pam3 (Figure 4B). In contrast, the OCR was not affected by individual stimulation with cIgG or Pam3, and only moderately increased upon co-stimulation (Figure 4C).

Next, we set out to investigate whether the amplification of the glycolytic response by FcγR-TLR cross-talk was also dependent on IRF5. While silencing of IRF5 did not affect the ECAR induced by individual stimulation with cIgG or Pam3, IRF5 silencing did inhibit the increased ECAR induced upon co-stimulation (Figure 4D). These data indicate that FcγR-TLR cross-talk amplifies the glycolytic response via IRF5.

To assess whether the increased glycolysis by FcγR-TLR cross-talk indeed contributes to the induction of cytokine responses, we stimulated moDCs in the presence of 2-deoxyglucose (2DG), which blocks glycolysis by inhibiting hexokinase activity (35). In line with previous findings, 2DG suppressed cytokine production induced by individual TLR stimulation (Figure 4E). In addition, 2DG also strongly suppressed cytokine production upon co-stimulation with cIgG and Pam3 (Figure 4E). Interestingly, while 2DG strongly impaired FcγR-TLR cross-talk-induced TNF protein production, blocking of glycolysis had very little effect on FcγR-TLR cross-talk-induced *TNF* gene transcription (representative donor Figure 4F, multiple donors Figure 4G). These data indicate that the glycolytic changes induced by FcγR-TLR cross-talk, although essential for protein production, have little effect on cytokine gene transcription.

Taken together, these data identify that IRF5 activation by FcγR-TLR cross-talk does not only enhance cytokine gene transcription, but also boosts translation through glycolytic reprogramming that together account for the strongly increased pro-inflammatory profile of moDCs activated by FcγR-TLR cross-talk.

## Discussion

**Figure 5:**
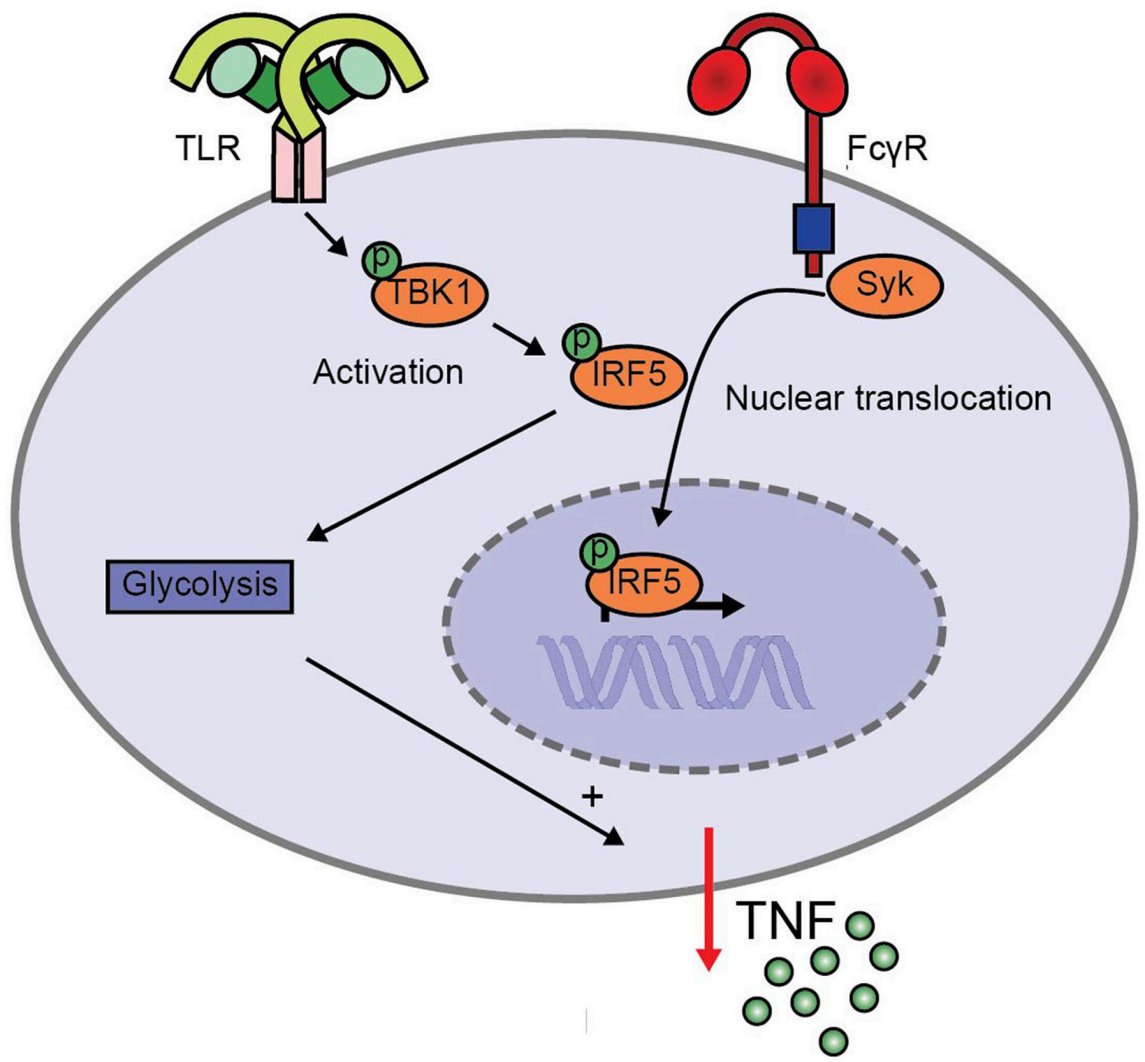
Model for enhanced TNF production upon FcγR-TLR cross-talk via IRF5. TLR stimulation induces TBK1/IKKε phosphorylation that leads to IRF5 phosphorylation, while FcγR signaling induces IRF5 nuclear translocation. Simultaneous activation of IRF5 by TLRs and FcγRs amplifies pro-inflammatory cytokine production in two ways. First, IRF5 increases cytokine gene transcription. Second, IRF5 increases the glycolytic rate, which amplifies cytokine production in a post-transcriptional manner.

FcγR-TLR cross-talk in human myeloid APCs is an important initiator of inflammation during both infection and autoimmunity (1, 2, 6, 8). However, the molecular mechanisms underlying this cross-talk are still largely unknown. Here, we identified a crucial role for IRF5, which is activated by two different pathways during FcγR-TLR co-stimulation to synergistically amplify pro-inflammatory cytokine production (schematically depicted in Figure 5). While TLR stimulation induces IRF5 phosphorylation, FcγR stimulation results in IRF5 nuclear translocation. In addition, we identified that during FcγR-TLR cross-talk IRF5 amplifies cytokine production in at least two different ways. First, IRF5 increases cytokine gene transcription. Second, IRF5 induces glycolytic reprogramming, which amplifies cytokine production in a post-transcriptional manner.

IRF5 is a transcription factor that was originally identified to be involved in type I interferon (IFN) production and antiviral responses. Over the last decade, multiple additional functions of IRF5 have been identified (24). Of these, the role of IRF5 in promoting transcription of pro-inflammatory cytokines such as TNF is the most pronounced (10–14). In addition, IRF5 expression has been identified as a marker to discriminate between subsets of macrophages, since IRF5 expression is higher in inflammatory macrophage subsets (12). Although IRF5 expression levels differ between different immune cells, our data indicate that IRF5 is required for FcγR-TLR cross-talk in various human APCs, including DCs and macrophages.

We identified that IRF5 promotes inflammation by both enhancing gene transcription and by inducing glycolytic reprogramming. IRF5 is known to enhance gene transcription of pro-inflammatory genes such as TNF by both directly binding to IFN-stimulated response element (ISRE) regions in the TNF promoter, and by forming a complex with other transcription factors, specifically NF-κB subunit p65 (13). Transcriptional activation of IRF5 is strictly regulated by different and independent post-translational modifications, to ensure initiation of appropriate immune response and prevent unrestrained inflammation. On one hand, IRF5 needs to be phosphorylated, which enables dimerization that is required for DNA binding (14, 25, 26, 28, 29). On the other hand, IRF5 needs to be translocated into the nucleus, which is achieved via K63-ubiquitination of IRF5 (25, 26, 36). Hence, either phosphorylation or ubiquitination individually are generally not sufficient for full IRF5 activation (24–26). Based on our findings and current literature we here propose a cooperation model of IRF5-dependent gene transcription upon FcγR-TLR cross-talk (schematically depicted in Figure 5). In this model, TLR stimulation induces TBK1/IKKε-dependent phosphorylation of IRF5, which is required for IRF5 activation. Additionally, FcγR stimulation induces Syk-dependent nuclear translocation of IRF5. Together, these two pathways cooperate leading to activated IRF5 inside the nucleus, thereby amplifying cytokine gene transcription.

How FcγRIIa triggering induces IRF5 nuclear translocation is still speculative, but it may result from Syk-dependent activation of an E3 ligase that induces K63-ubiquitination of IRF5. Interestingly, Syk has been previously coupled to IRF5 activation, which was indeed independent of IRF5 phosphorylation (24, 37). In this regard, a relevant candidate E3 ligase is TRAF6 (38, 39), which has previously been identified to K63-ubiquinate IRF5 (36). Interestingly, also TRAF6 activation by Syk has been described to be dependent on K63-linked ubiquitination (40). Another candidate is Pellino-1, which additionally provides a connection between K63-ubiquitination of IRF5 and glucose metabolism (41).

In addition to increasing gene transcription, we identified that FcγR-TLR cross-talk also induces glycolytic reprogramming by IRF5. Interestingly, this finding corroborates a recent study by Hedl et al., which shows that IRF5 regulates the glycolytic rate in human and murine macrophages (34). IRF5 increases the glycolysis upon NLR stimulation via activation of the kinase Akt2, which upregulates the transcription of various glycolytic genes (34). However, remarkably, the phosphorylation of Akt2, which is essential for Akt2 activation, is independent of IRF5 phosphorylation (34), suggesting that also other posttranslational modifications of IRF5 are required for increasing glycolysis. Since FcγR stimulation induces IRF5 nuclear translocation, which is dependent on K63-ubiquitination (36, 41), the increased glycolysis by FcγR-TLR cross-talk may therefore depend on multiple posttranslational modifications of IRF5, which ultimately lead to increased Akt2 activation and glycolysis.

FcγRs such as FcγRIIa signal through an ITAM sequence in the cytoplasmic tail, which is a common signaling module used by a variety of receptors, including B cell receptors and T cell receptors, and other members of the Fc receptor family (42, 43). Interestingly, cross-talk with TLRs has previously been described for various other Fc receptor family members, including FcαRI (33, 44) and FcεRI (45, 46). In addition, Fc receptors have been shown to not only amplify cytokine responses induced by TLRs, but also by several other receptors such as NLRs, C-type lectins, IL-1R, and IFNγR (2, 33, 44). The fact that the cross-talk of different Fc receptors with various PRRs and cytokine receptors in different cell types all amplify pro-inflammatory cytokines in a similar manner suggests that the identified pathway may be a general mechanism of synergy between ITAM signaling receptors and PRRs, analogous to the previously described collaboration between the ITAM signaling module and JAK-STAT signaling pathways (42).

FcγR-TLR cross-talk provides protective immunity against various pathogens including bacteria and viruses (1, 3, 47), but is detrimental in various autoimmune diseases, since it strongly promotes the production of pathogenic pro-inflammatory cytokines (6, 8). Interestingly, IRF5 activation is also tightly associated with various chronic inflammatory disorders (17, 18, 21, 22). In addition, disease-associated *IRF5* polymorphisms have previously been shown to dramatically affect cytokine production by myeloid immune cells by both increasing gene transcription and glycolysis (34, 48). Disease-associated *IRF5* polymorphisms are generally associated with higher IRF5 expression, but some polymorphisms also give rise to novel IRF5 isoforms (49). For future research, it would be very interesting to determine whether disease-associated IRF5 polymorphisms also promote cytokine production by enhancing FcγR-TLR cross-talk. In addition, targeting of IRF5, or its upstream activators such as TBK1/IKKε, may open a new avenue for therapeutic intervention (22, 50).

Taken together, we identified IRF5 as a key component of FcγR-TLR cross-talk in human antigen-presenting cells. Our data strengthen the concept of a powerful pro-inflammatory role of IRF5 through amplification of gene transcription and metabolic reprogramming. Because undesired activation by autoantibodies contributes to the pathogenesis of various chronic inflammatory disorders, targeting of FcγR-TLR signaling may be a valuable tool to suppress inflammation in diseases such as RA, systemic lupus erythematous (SLE), and inflammatory bowel disease (IBD).

## Author Contributions

JdD: conceptualization; WH, MN, LV, MK, BE, and JdD: methodology; WH, MN, LV, LS, IH, BE, and JdD: investigation; WH, LV, and JdD: writing the original draft; MK, DB, BE, and JdD: reviewing and editing the manuscript.

### Conflict of Interest Statement

DB is also an employee of Union Chimique Belge. The remaining authors declare that the research was conducted in the absence of any commercial or financial relationships that could be construed as a potential conflict of interest.
